# Epidemiology of central-line–associated bloodstream infection mortality in Canadian NICUs before and after 2017

**DOI:** 10.1017/ash.2023.287

**Published:** 2023-09-29

**Authors:** Maria Spagnuolo, Anada Silva, Jessica Bartoszko, Linda Pelude, Blanda Chow, Jeannette Comeau, Chelsey Ellis, Charles Frenette, Lynn Johnston, Kevin Katz, Joanne Langley, Bonita Lee, Santina Lee, Marie-Astrid Lefebvre, Allison McGeer, Dorothy Moore, Senthuri Paramalingam, Jennifer Parsonage, Donna Penney, Caroline Quach, Michelle Science, Stephanie Smith, Kathryn Suh, Jocelyn Srigley

## Abstract

**Background:** The Canadian Nosocomial Infection Surveillance Program (CNISP) observed increased mortality among neonatal intensive care unit (NICU) patients with central-line–associated bloodstream infection (CLABSI) starting in 2017. In this study, we compared NICU patients with CLABSIs before and after 2017, and quantified the impact of epidemiological factors on 30-day survival. **Methods:** We included 1,276 NICU patients from 8–16 participating CNISP hospitals from the pre-2017 period (2009–2016) and the post-2017 period (2017–2022) using standardized definitions and questionnaires. We used Cox regression modeling to assess the impact of age at date of positive culture, sex, birthweight, CLABSI microorganism, region of the country, and surveillance period (before 2017 vs after 2017) on time to 30-day all-cause mortality from date of positive culture. Gestational age was not available for this analysis. We reported model outputs as hazard ratios with 95% CIs. **Results:** In total, 769 (60%) NICU CLABSIs were reported in the pre-2017 period and 507 (40%) in the post-2017 period. The 30-day all-cause mortality rate was 8% (n = 100 of 1,276) overall, and significantly higher after 2017 (12%, n = 61 of 507) than before 2017 (5%, n = 39 of 769) (*P* < .001).

During the post-2017 period, cases were significantly younger: 16 days (IQR, 9–33) versus 21 days (IQR, 11–49) (*P* = .002). Median days from ICU admission to infection were shorter: 14 (IQR, 8–31) versus 19 (IQR, 10–41) (*P* < .001). More gram-negative CLABSIs were identified (29% vs 24%; *P* = .040) and fewer gram-positive CLABSIs were identified (64% vs 72%; *P* = .006) compared to the pre-2017 period. Mortality was higher in CLABSIs caused by gram-negative bacteria (15%, n = 50 of 328) than gram-positive bacteria (4.4%, n = 39 of 877) (*P* < .001), and mortality was higher in neonates with birthweight <1,000 g (11%, n = 71 of 673) compared to those weighing ≥1,000 g (5%, n = 28 of 560) (*P* < .001).

Adjusting for all other factors, survival modeling indicated that NICU CLABSIs identified in the post-2017 period had 2.12 (95% CI, 1.23–3.66) times the hazard ratio of 30-day all-cause mortality compared to those before 2017 (*P* < .006). Those identified with a gram-positive bacterium had a 0.28 hazard ratio (95% CI, 0.12–0.65) of 30-day mortality compared to those with a gram-negative bacterium or fungus (*P* = .003). In the fully adjusted model, age, sex, and birthweight were not significantly associated with NICU CLABSI survival. **Conclusions:** NICU patients with CLABSIs had significantly higher all-cause mortality between 2017–2022 compared to 2009–2016, and those who acquired gram-positive–associated CLABSIs had improved survival compared to other organisms. Further work is needed to identify and understand factors driving the increased mortality among NICU CLABSI patients from 2017–2022.

**Disclosures:** None

